# Telomere length in Chernobyl accident recovery workers in the late period after the disaster

**DOI:** 10.1093/jrr/rru060

**Published:** 2014-07-11

**Authors:** Jelena Reste, Gunda Zvigule, Tija Zvagule, Natalja Kurjane, Maija Eglite, Natalija Gabruseva, Dace Berzina, Juris Plonis, Edvins Miklasevics

**Affiliations:** 1 Institute of Occupational Safety and Environmental Health, Riga Stradins University, Dzirciema Street 16, Riga, LV-1007, Latvia; 2 Centre of Occupational and Radiological Medicine, Pauls Stradins Clinical University Hospital, Pilsonu Street 13, Riga, LV-1002, Latvia; 3 Institute of Oncology, Riga Stradins University, Dzirciema Street 16, Riga, LV-1007, Latvia

**Keywords:** aging-associated diseases, cancer, Chernobyl nuclear power plant accident, clean-up worker, protracted radiation exposure, telomere length

## Abstract

The outcome of the Chernobyl nuclear power plant (CNPP) accident was that a huge number of people were exposed to ionizing radiation. Previous studies of CNPP clean-up workers from Latvia revealed a high occurrence of age-associated degenerative diseases and cancer in young adults, as well as a high mortality as a result of cardiovascular disorders at age 45–54 years. DNA tandem repeats that cap chromosome ends, known as telomeres, are sensitive to oxidative damage and exposure to ionizing radiation. Telomeres are important in aging processes and carcinogenesis. The aim of this study was to investigate the long-term effect of protracted ionizing radiation exposure on telomere length in CNPP clean-up workers. Relative telomere length (RTL) was measured in peripheral blood leukocytes of 595 CNPP clean-up workers and 236 gender- and age-matched controls using real-time quantitative polymerase chain reaction (q-PCR). Close attention was paid to participation year and tasks performed during the worker's stay in Chernobyl, health status, and RTL differences between subgroups. Telomere shortening was not found in CNPP clean-up workers; on the contrary, their RTL was slightly greater than in controls (*P* = 0.001). Longer telomeres were found in people who worked during 1986, in those undertaking ‘dirty’ tasks (digging and deactivation), and in people with cancer. Shorter telomeres appeared frequently in those with cataract, osteoporosis, atherosclerosis, or coronary heart disease. We conclude that the longer telomeres revealed in people more heavily exposed to ionizing radiation probably indicate activation of telomerase as a chromosome healing mechanism following damage, and reflect defects in telomerase regulation that could potentiate carcinogenesis.

## INTRODUCTION

Ionizing radiation health effects have received much attention in recent decades due to the growing usage of nuclear power as an energy source and the occurrence of a number of recent serious accidents involving the release of radioactive elements into the environment. Nuclear power plant damage can create radioactive pollution over a wide area and expose a huge number of people to ionizing radiation, as was seen in the Chernobyl and Fukushima accidents. The irradiation doses received by victims of such accidents in most cases do not exceed 1 Gy (e.g. the Chernobyl disaster) [1, 2], however in A-bomb explosions high doses are inflicted [3]. The effect of high doses can be clearly seen either immediately or within a short time after exposure in the form of acute radiation sickness, whereas low-dose radiation exposure is not so evident and initially does not show any visible clinical symptoms [4]. Much uncertainty still remains about the effects of low-dose ionizing radiation on human health. Almost the only clearly proven effect of long-term low-dose radiation exposure is cancer development [5]. A number of studies that have been conducted to investigate the effects of exposure to ionizing radiation on health suggest probable irradiation-dependent development of non-cancer diseases such as coronary heart disease, myocardial infarction, stroke, arterial hypertension, mental disorders, cataract, etc., but evidence is limited [5, 6]. Many of these diseases are aging-associated. Some signs of premature aging have been shown in studies of people exposed to radiation [7, 8], but the precise mechanisms for this probable acceleration of aging processes still remain unclear.

Telomeres play an important role in cell aging. Telomeres are DNA tandem repeats of six bases, (TTAGGG)*n*, and are located at the ends of chromosomes. They cap chromosome ends and protect cells against end-to-end fusion between chromosomes and against entering apoptosis. Telomere shortening in human somatic cells occurs with each cell division [9]. In normal conditions, after a certain limited number of divisions (known as the ‘Hayflick cell division limit’ [10, 11]) cells enter irreversible cell cycle arrest, which finishes with cell death [12]. Telomere shortening is thought to be an ‘internal clock’ which terminates a cell's lifespan, because critically short telomeres cannot protect chromosome ends from fusion and may initiate replicative senescence and apoptosis [13]. On the other hand, the enzyme telomerase works against telomere shortening [14, 15]. Telomerase adds specific telomeric DNA repeats, thus compensating the loss of telomere end sequences during cell divisions [16]. The expression of telomerase is activated by DNA damage as well as through a number of internal mechanisms [17]. High activity of telomerase has been observed in actively dividing cells and in cancer cells. Contrariwise to normal somatic cells, cancer cells may develop immortality owing to their high telomerase activity [18]. Telomeres are rich in guanine and highly susceptible to oxidative damage [19 –21]. At the same time, DNA damage repair mechanisms in the telomere zone are not as effective as in other parts of the DNA [22]. Taking into account that ionizing radiation may damage DNA, telomeres may be particularly sensitive to radiation-induced damage [23]. Information from literature reviews on changes in telomere length after ionizing radiation exposure is limited and ambiguous. Several studies show no difference in telomere length after radiation exposure [24]. Some studies show telomere elongation after radiation-induced DNA damage and suggest that telomere elongation might be a healing mechanism of chromosomes after DNA damage [25, 26]. These results have been obtained mostly through experiments on animal models; evidence from exposed humans is limited [27].

The present paper represents the results of telomere length measurements in peripheral blood leukocytes (PBLs) of Chernobyl nuclear power plant (CNPP) clean-up workers from Latvia ∼23 years after participation in clean-up works. Previously reported studies on morbidity in CNPP clean-up workers from Latvia [28–33] have revealed a high occurrence of aging-associated degenerative diseases and malignant neoplasms in relatively young accident liquidators, as well as high mortality from cardiovascular disorders in the age group 45–54 years [30]. Taking into account that previous studies about radiation exposure effects on telomere length were mostly made in animal or cell models shortly after exposure to high doses of radiation [24 –26] rather than investigating the long-term effects, the aim of our study was exploration of telomere length changes in exposed humans a long time after protracted exposure to small doses of radiation from internally deposited radionuclides. In the current study, we test the hypothesis that telomere length may be altered in people with previous long-term ionizing radiation exposure compared with an age- and gender-matched, non-exposed population. Close attention is paid to the current health status of CNPP clean-up workers, their participation year and tasks performed during their stay in Chernobyl, and telomere length differences between subgroups based on these factors.

## MATERIALS AND METHODS

The current study was designed to evaluate the effect of previous long-term ionizing radiation exposure on telomere length in PBLs of CNPP clean-up workers. We performed a cross-sectional study ∼23 years after work in Chernobyl. In total, relative telomere length (RTL) was measured in 831 males. During the study, blood samples and the health status data of 595 CNPP clean-up workers from Latvia and a population of 236 gender- and age-matched control people were investigated. The CNPP clean-up workers were divided into several subgroups, and telomere length was assessed comparing subgroups with each other and with the control group. The study was approved by the Regional Committee for Medical Research Ethics. Informed written consent was received from every study participant.

### Study population

Study participants were randomly selected from the CNPP clean-up workers examined in the Centre of Occupational and Radiological Medicine Inpatient and Outpatient Departments of the Pauls Stradins Clinical University Hospital (‘the Centre’) in Riga. The selection criteria for exposed participants were people: (i) who have survived at least 20 years after participation in clean-up works, (ii) for whom there is documented and proven participation in CNPP clean-up works at any time during the period from 26 April 1986 till the end of 1991, (iii) with known data about current health condition, and (iv) who voluntarily participated and provided signed, informed written consent. Exclusion criteria were: (i) refusal of voluntary participation in the study, and (ii) people with extremely severe somatic pathology preventing them from coming to the Centre. All study participants were males. Most of these men participated in CNPP clean-up works for a period of 1–3 months. The group of CNPP clean-up workers studied was a subset of the 6000 Latvian inhabitants sent by the Soviet Union to Chernobyl to combat the consequences of the disaster.

The main tasks allocated during the work in Chernobyl were: deactivation of contaminated radioactive objects, soil decontamination by digging and mechanical removal, transportation of people and contaminated materials, construction, and encampment supporting activities (food supply, etc.). None of the study participants developed acute radiation sickness symptoms either in Chernobyl or after returning to Latvia. The CNPP clean-up workers have undergone clinical examination in the Centre at least once every 2–3 years since 1994, thus these people have data about their health status for greater than a 15-year period. Additional information on health status, participation year in clean-up works, tasks performed during their stay in Chernobyl, and documented irradiation dose was obtained from the Latvian State Register for people exposed to ionizing radiation in Chernobyl (‘the Register’). All information on diseases revealed by regular examination was stored in the Register using the International Classification of Diseases, 10th Revision (ICD-10). Only 58% of study participants had information about their dose of radiation exposure. The documented dose range was 0.8–383.0 mSv, the median dose was 120 mSv, and the interquartile range (IQR range, i.e. 25th and 75th percentiles) was 89–190 mSv. Considering the inaccuracy in the radiation dose assessment in Chernobyl (only external irradiation was measured for calculations of documented doses [7, 34, 35]; internal irradiation was not evaluated), documented doses were considered unreliable. Therefore, participation time and tasks performed during stay in Chernobyl were employed for the evaluation of radiation exposure in the CNPP clean-up workers.

The analysed CNPP workers' ‘health status data’ included the presence of cataract, malignant neoplasms, insulin-independent diabetes, thyroid gland benign diseases, benign prostate hyperplasia, osteoporosis, atherosclerosis, coronary heart disease and angina pectoris due to obliterative atherosclerosis confirmed by medical examinations. The prevalence of these disorders in the study participants (divided into subgroups) compared with the general population of CNPP clean-up workers from Latvia is shown in the Supplementary Data. The prevalence of certain disorders in the study group was slightly higher than in the general population of CNPP clean-up workers from Latvia due to the thorough medical health examinations performed during their most recent visits to the Centre; the remaining CNPP clean-up workers visit the Centre more rarely, and some diseases may be undiagnosed.

Gender- and age-matched healthy blood donors were chosen for the control group. Individuals included in the control group were healthy people living in Latvia who have not previously been exposed to excessive ionizing radiation, other than natural background radiation and rare small medical X-ray examinations.

For achieving a more precise assessment the impact of radiation on health, it was decided to separate CNPP clean-up workers with relatively high radiation exposure from less-exposed accident liquidators and to compare telomere length in these groups. For comparative analysis, ‘CNPP clean-up workers’ were divided into several subgroups according to:

- year of participation in clean-up works in Chernobyl: during 1986 (*n* = 302), or from 1987 till 1991 (*n* = 271);
- tasks performed during clean-up works: people who performed deactivation of radioactive objects and digging, with high risk of being contaminated with radioactive materials and a potentially higher dose of irradiation (*n* = 231); or people who performed other tasks (drivers, builders, cooks, etc.) (*n* = 353);
- people who participated in 1986 and performed deactivation and digging tasks (i.e. exposed to higher radiation doses) were identified as a high-risk group (*n* = 140); all other people were graded as a low-risk group (*n* = 444).

### Blood sample collection and DNA extraction

Blood samples were collected from CNPP clean-up workers between 2010 and 2012, i.e. ∼23 years (range, 19–26 years) after their work in Chernobyl during the period 1986–1991. Blood samples were obtained from the cubital vein in 3-ml sterile vacutainers coated with EDTA (ethylenediaminetetraacetic acid). Vacutainers were labelled and immediately transported to the laboratory within 24 h. Genomic DNA was isolated from whole blood using a FlexiGene Kit (205) (Qiagen, Dusseldorf, Germany) according to the manufacturer's instructions. Acquired DNA quality was tested by 1% agarose gel electrophoresis. DNA concentration was measured by NanoDrop Spectrophotometer ND-1000. DNA samples were stored in 10 mM Tris-Cl (tris(hydroxymethyl)amino methane) buffer solution at pH = 8.5 and +4°C temperature until the time of assay.

### Telomere length measurement

RTL was measured by real-time quantitative polymerase chain reaction (q-PCR) using the approach described by Cawthon [36]. DNA samples were diluted to a concentration of 10.0 ng/μl prior to performing real-time q-PCR. For reference, a DNA sample from a healthy age-matched male without malignancies in anamnesis and not previously exposed to excessive ionizing radiation (other than natural background radiation and rare small medical X-ray examinations) was selected. A dilution line of concentrations 50.0 ng/μl, 16.8 ng/μl, 5.25 ng/μl and 1.82 ng/μl was made from the reference DNA sample. Reference samples were amplified in each run. The PCR mixture was prepared as follows: 2.5 μl of × 10 Taq polymerase buffer (Takara, Japan), 0.2 μl of 10 mM dNTP mixture (Takara, Japan), 2.25 μl of em1109 primer (10 pM), 2.25 μl of em1110 primer (10 pM), 2.25 μl of em1111 primer (10 pM), 2.25 μl of em1112 (10 pM) primer, 2 μl of 5 nM SYBR Green (Invitrogen, USA), 0.5 U of Taq polymerase (SPEED STAR, Takara, Japan) and 9.3 μl of distilled water. To each well, 2 μl of diluted DNA sample and 23 μl of PCR mixture were added. The specific telomere and single-copy gene primers were supplied by Integrated DNA Technologies (IDT), Inc., USA. The primer sequences were as follows:

telg em1109, 5′ ACACTAAGGTTTGGGTTTGGGTTTGGGTTTGGGTTAGTGT 3′,

telc em1110, 5′ TGTTAGGTATCCCTATCCCTATCCCTATCCCTATCCCTAACA 3′,

albu em1111, 5′ CGGCGGCGGGCGGCGCGGGCTGGGCGGAAATGCTGCACAGAATCCTTG 3′, and

albd em1112, 5′ GCCCGGCCCGCCGCGCCCGTCCCGCCGGAAAAGCATGGTCGCCTGTT 3′.

Each sample was run in triplicate. The PCR plates were inserted into an Rotor-Gene 6000 amplificator (Corbett, USA). The thermal condition programme for real-time q-PCR consisted of three steps: the first one—15 min at 95°C; the second—two cycles at 94°C for 15 s and at 49°C for 15 s; the third—32 cycles at 94°C for 15 s, 62°C for 10 s, 74°C for 15 s with signal acquisition, 84°C for 10 s and 88°C for 15 s with signal acquisition. The cycle threshold (C_t_) value of the signal acquired at 74°C denoted the product of telomere amplification, but the C_t_ value of the signal acquired at 88°C showed the single-copy gene product. Normalisation and analysis of measurements were processed by Rotorgene 6000 series software 1.7.94 (Corbett, USA). Reference lines were obtained from diluted reference samples, single-copy gene and from telomere amplification results. C_t_ values of single-copy gene (S) and telomere (T) signals were used for calculation of the T/S ratio (equivalent to the RTL), which is proportional to individual telomere length. The T/S ratio was expressed in conditional units (U). All procedures were undertaken in a blinded manner without knowledge of the personal information related to the sample being tested.

### Statistical analysis

Appropriate statistical methods were employed according to the shape of data distribution. The models were adjusted for age. For achieving precise age-effect evaluation, data were weighted by age before analysis using an integrated weighting approach. For performing detailed analysis, outliers were excluded from calculations. The association of RTL with age was evaluated using Spearman's correlation analysis. For comparing subgroups with each other, independent samples *t*-tests and Mann–Whitney tests were used, and odds ratios (ORs) and the 95% confidence intervals (CIs) were calculated. The significance level was set at 0.05. All calculations were completed using Microsoft Excel and IBM SPSS Statistics Version 20.

## RESULTS

**Fig. 1.**
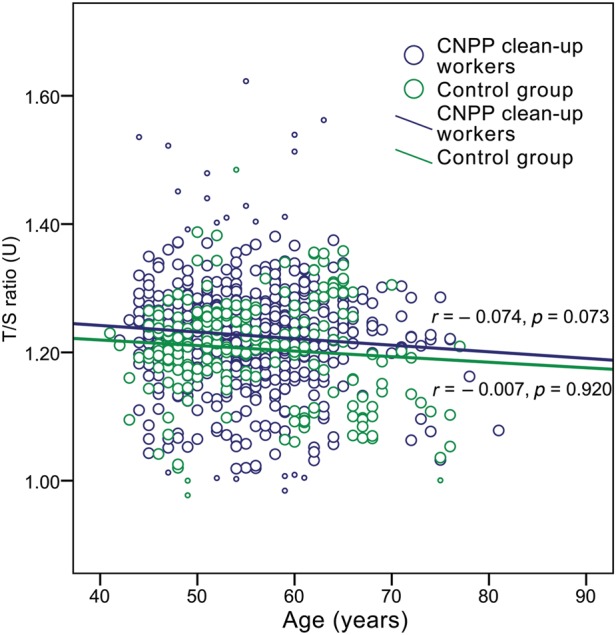
Relative telomere length (T/S ratio) in Chernobyl accident recovery workers and controls by age, indicating trend lines for each group (Spearman's correlation coefficient *r* and *P*-value for each group are shown; small circles indicate excluded outliers).

**Fig. 2.**
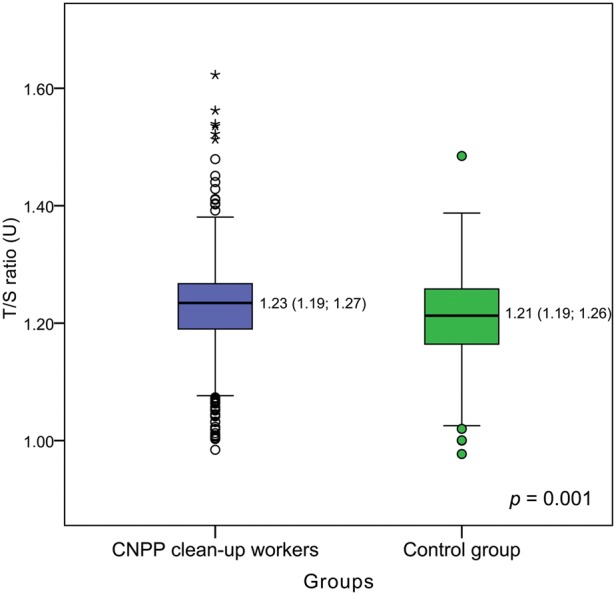
Boxplots of relative telomere length (T/S ratio) in Chernobyl accident recovery workers and the gender- and age-matched control group (median value and interquartile range are shown; circles indicate outliers, asterisks indicate extreme outliers).

As mentioned earlier, the aim of the current study was to find out the effect of previous long-term ionizing radiation exposure on telomere length in human PBLs. In total, the telomere length of 831 people was evaluated. The results of the RTL measurements by q-PCR in exposed and non-exposed groups are displayed in Fig. 1 and Fig. 2. Large interindividual variability, even among people of the same age, was observed in both groups. A slightly higher RTL was observed in CNPP clean-up workers than in the control group, and the difference appeared to be statistically significant (*P* = 0.001). The median T/S ratio in Chernobyl accident workers was 1.23 U (IQR, 1.19–1.27 U) vs 1.21 U (IQR, 1.16–1.26) in controls. Despite the relatively larger number of exposed people (*n* = 595) compared with controls (*n* = 236), the age distribution of the CNPP clean-up workers in the study group did not differ significantly from that of the control group (an independent samples *t*-test did not reveal significant differences, *t* = −1.084, *P* = 0.279). The mean age of the CNPP clean-up workers was 54.95 ± 6.78 years (age range, 42–81 years), and in the control group was 55.55 ± 8.07 years (age range, 44–78 years).

The results of the RTL measurements for the various subgroups of the CNPP clean-up workers and the control group are summarised in Table 1. Taking into consideration participation year in the Chernobyl clean-up works, there were no significant differences in the T/S ratio between the 1986 and the 1987–1991 participants (*P* > 0.05), but the median T/S ratio of both subgroups was still higher than that of the controls (*P* < 0.01). In addition, the longest telomeres were found in the 1986 participants.

**Table 1.** Comparison of relative telomere length (median T/S ratio (IQR) and mean ranks of T/S ratio) between CNPP accident clean-up workers' subgroups and the control group

Comparable groups	Numbers of participants in corresponding groups	Median T/S ratio (IQR) in corresponding groups (U)	Mean ranks^a^ of T/S ratio in corresponding groups	*P*-value^b^
1986/controls	302/236	1.24 (1.18, 1.27)/1.21 (1.16, 1.26)	427.36/363.28	0.004
1987–1991/controls	271/236	1.23 (1.19, 1.26)/1.21 (1.16, 1.26)	270.04/235.58	0.008
1986/1987–1991	302/271	1.24 (1.18, 1.27)/1.23 (1.19, 1.26)	291.15/282.37	0.526
Deactivation tasks/controls	231/236	1.24 (1.20, 1.27)/1.21 (1.16, 1.26)	445.79/366.94	0.001
Other tasks/controls	353/236	1.23 (1.19, 1.27)/1.21 (1.16, 1.26)	309.41/273.45	0.012
Deactivation tasks/other tasks	231/353	1.24 (1.20, 1.27)/1.23 (1.19, 1.27)	305.31/284.12	0.138
High-risk group/controls	140/236	1.24 (1.19, 1.28)/1.21 (1.16, 1.26)	445.54/366.94	0.002
Low-risk group/controls	444/236	1.23 (1.19, 1.27)/1.21 (1.16, 1.26)	356.79/309.85	0.003
High-risk group/low-risk group	140/444	1.24 (1.19, 1.28)/1.23 (1.19, 1.27)	305.78/288.31	0.285

^a^The Mann–Whitney test was used for calculations. ^b^The *P*-value characterizes differences between selected groups, the

T/S ratio indicates the proportion of C_t_ values of telomere (T) and single-copy gene (S) signals acquired in q-PCR (corresponds to relative telomere length).

IQR = interquartile range (25th and 75th percentiles).

When we evaluated the T/S ratio difference between the various task performers, the deactivation and digging workers were found to have minimally longer telomeres compared with workers who carried out other tasks (*P* = 0.138), even though the age structure of the two subgroups did not differ markedly (*P* = 0.777). Comparing these subgroups with the control group revealed a significantly longer T/S ratio in both task groups compared with the control group (*P* < 0.05); moreover ‘dirty’ task performers had noticeably longer telomeres than controls did (*P* = 0.001).

After grouping the workers into ‘risk groups’ according to participation year and tasks performed, longer telomeres were found in the high-risk group (participants who worked in 1986 and performed ‘dirty’ tasks) compared with the low-risk group (participants who worked in 1987–1991 at other kinds of tasks), but the difference was not statistically significant (*P* = 0.285). However, the control group was demonstrated to have significantly shorter telomeres in non-exposed people compared with both risk groups of CNPP accident workers (*P* < 0.01).

**Fig. 3.**
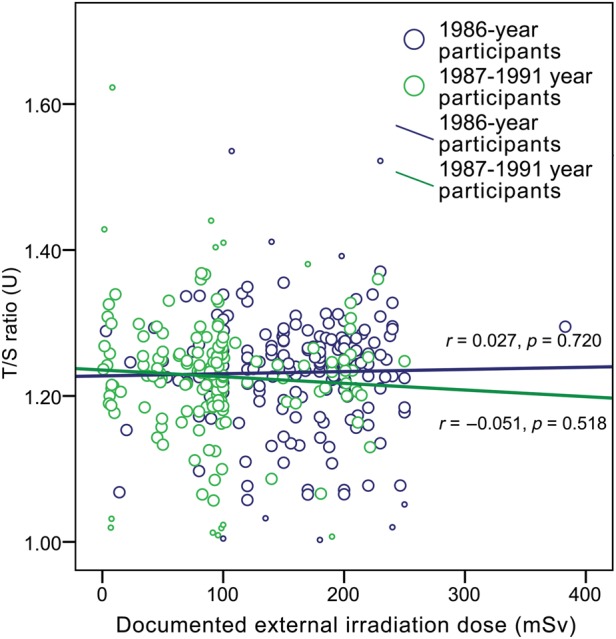
The effect of irradiation dose (documented external irradiation dose shown) on relative telomere length (T/S ratio) in CNPP clean-up workers separated according to year of participation in clean-up works (Spearman's correlation coefficient *r*, *P*-value and trend line indicated for each group; small circles indicate excluded outliers).

**Fig. 4.**
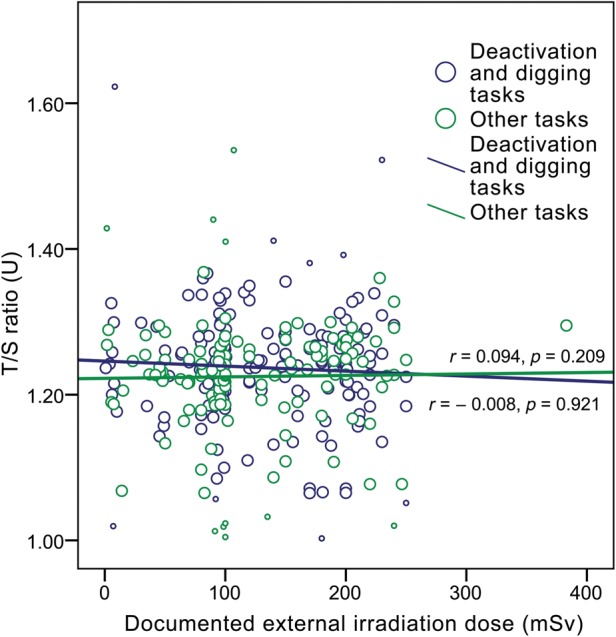
The effect of irradiation dose (documented external irradiation dose shown) on relative telomere length (T/S ratio) in CNPP clean-up workers separated according to tasks performed during stay in Chernobyl (Spearman's correlation coefficient *r*, *P*-value and trend line indicated for each group; small circles indicate excluded outliers).

Despite previously mentioned T/S ratio differences between the subgroups, the analysis of the association between the RTL and the documented dose of radiation exposure revealed only a very weak and insignificant correlation between telomere length and the recorded dose in CNPP clean-up workers (for the total CNPP clean-up workers group, the Spearman's correlation coefficient, *r_s_*, was 0.051, *P* = 0.349; Fig. 3 and Fig. 4).

**Fig. 5.**
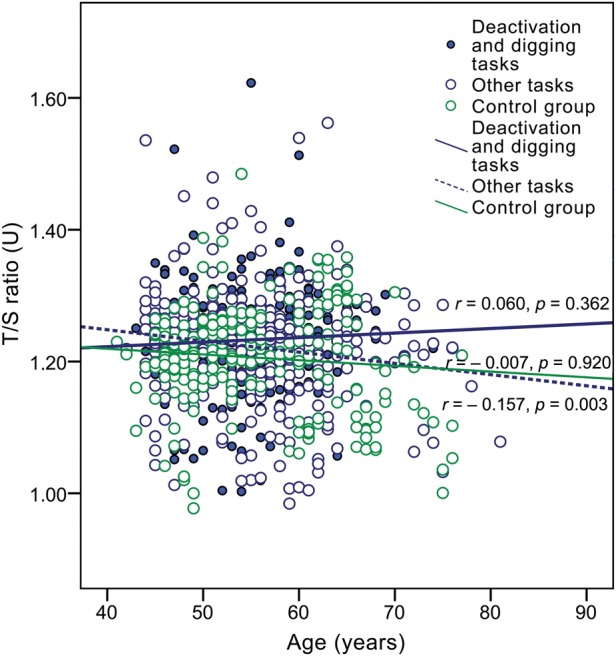
The effect of different types of job in Chernobyl on relative telomere length (T/S ratio) according to age in CNPP accident recovery workers compared with controls (Spearman's correlation coefficient *r*, *P*-value and trend lines indicated for each group).

For assessment of the effect of aging on telomere length, Spearman's correlation analysis was carried out. A very weak negative, statistically insignificant correlation between T/S ratio and documented age of CNPP clean-up workers was observed (Spearman's coefficient *r_s_* = −0.074, *P* = 0.073). At the same time, such correlation was not found in the control group (*r_s_* = −0.007, *P* = 0.920) (see Fig. 1). The effect of age on telomere length did not differ significantly between the 1986 and 1987–1991 participants and showed a similar trend to that found in the total CNPP workers group. A more notable difference between the CNPP subgroups and the control group was revealed after dividing the CNPP accident workers according to tasks performed (see Fig. 5). A very slight insignificant telomere lengthening with age was observed in those who performed ‘dirty’ tasks (*r_s_* = 0.060, *P* = 0.362); however, in other task performers, a statistically significant weak negative correlation of T/S ratio was found with increasing age (*r_s_* = −0.157, *P* = 0.003). Analogous coherence was observed in high- and low-risk CNPP workers: in the high-risk group a statistically non-significant, very weak positive correlation of T/S ratio with age was identified (*r_s_* = 0.104, *P* = 0.222), whereas in the low-risk group, a statistically significant, weak negative correlation was observed (*r_s_* = −0.142, *P* = 0.003).

Detailed analysis of the RTL in CNPP clean-up workers with respect to health disturbances developed after returning from Chernobyl revealed major differences. The results are summarised in Table 2, in which the RTL of CNPP clean-up workers suffering from a range of particular diseases was compared with the RTL in people without the particular diseases. As is evident from the table, the longest telomeres were observed in CNPP clean-up workers with malignancies (median T/S ratio, 1.25 U (IQR: 1.21–1.28)). In people with malignant neoplasms, the telomere length was longer than in people without malignancies (*P* = 0.053), regardless of the slightly older age of people with neoplasms—the mean age of oncologic patients was 58.33 ± 6.22 years, whereas in people without neoplasms it was 54.60 ± 6.72 years. After additional detailed adjusting of results for age, a significant difference was found (*P* < 0.001). On the other hand, the shortest telomeres were found in CNPP clean-up workers who suffered from chronic coronary heart disease and angina pectoris (median T/S ratio, 1.22 U (IQR: 1.18–1.26)), as well as in people with aging-associated illnesses such as atherosclerosis, osteoporosis and insulin-independent diabetes; however, compared with people without these disorders, the difference was not statistically significant. Taking into account the effect of malignancies on telomere length, people with malignancies were excluded from the analysis, but, irrespective of this, telomere length in CNPP clean-up workers still remained statistically significantly longer than in the control group (*P* = 0.003).

**Table 2.** Differences in relative telomere length (median T/S ratio (IQR) and mean ranks of T/S ratio) among CNPP accident clean-up workers suffering from particular diseases compared with liquidators without each particular disease

Comparable groups	Numbers of participants in corresponding groups	Median T/S ratio (IQR) in corresponding groups (U)	Mean ranks^a^ of T/S ratio in corresponding groups	*P*-value^b^
Malignant neoplasm present/absent	52/514	1.25 (1.21, 1.28)/1.23 (1.19, 1.27)	325.35/279.27	0.053
Insulin-independent diabetes present/absent	56/510	1.22 (1.15, 1.27)/1.23 (1.19, 1.27)	260.27/286.05	0.263
Benign thyroid gland diseases present/absent	276/290	1.23 (1.19, 1.27)/1.23 (1.19, 1.27)	285.62/281.48	0.763
Benign prostate hyperplasia present/absent	100/466	1.23 (1.19, 1.27)/1.23 (1.19, 1.27)	286.83/282.79	0.823
Osteoporosis present/absent	41/525	1.22 (1.19, 1.26)/1.23 (1.19, 1.27)	263.37/285.07	0.413
Senile cataract present/absent	221/345	1.23 (1.17, 1.27)/1.23 (1.20, 1.27)	269.83/292.26	0.112
Atherosclerosis present/absent	90/476	1.22 (1.19, 1.27)/1.24 (1.19, 1.27)	268.62/286.31	0.347
Chronic coronary heart disease and angina pectoris present/absent	145/421	1.22 (1.18, 1.26)/1.24 (1.19, 1.27)	260.95/291.27	0.054

^a^The Mann–Whitney test was used for calculations. ^b^The *P*-value characterizes differences between selected groups, the

T/S ratio indicates the proportion of C_t_ values of telomere (T) and single-copy gene (S) signals acquired in q-PCR (corresponds to relative telomere length).

IQR = interquartile range (25th and 75th percentiles).

For evaluation of the impact of telomere length on the development of various disorders, the total group of CNPP clean-up workers was divided according to whether the RTL was above or below the median value of the T/S ratio (1.23 U). T/S ratios above the median value were designated ‘long telomeres’, and those below were designated ‘short telomeres’. The frequency and OR for disease development was estimated for each group so as to compare an association with long telomeres versus short telomeres. The results of the calculations are indicated in Table 3. It is apparent that people with long telomeres had almost twice the risk of developing a malignant neoplasm (OR 1.88; 95% CI: 1.02, 3.45) compared with people with short telomeres. At the same time, short telomeres predisposed CNPP accident workers for getting myocardial infarction, angina pectoris, and chronic coronary heart disease, although the difference was not statistically significant (*P* > 0.05). People with short telomeres were also prone, albeit at lower distinct, to get osteoporosis, senile cataract, insulin-independent diabetes and benign prostate hyperplasia than participants with long telomeres. The risk of developing benign thyroid diseases was similar in both groups.

**Table 3.** Odds for development of a certain disorder in CNPP clean-up workers with a relative telomere length above the median value (1.23 U) of the T/S ratio compared with the group with a relative telomere length below the median value (OR and 95% CI)

Pathology	Number of cases in long telomere group	Number of cases in short telomere group	OR^a^	95% CI^b^
Malignant neoplasm	35	17	1.88	1.02–3.45
Insulin-independent diabetes	28	28	0.85	0.48–1.48
Benign thyroid gland diseases	148	128	1.00	0.72–1.39
Benign prostate hyperplasia	51	49	0.88	0.57–1.36
Osteoporosis	19	22	0.73	0.39–1.38
Senile cataract	110	111	0.78	0.55–1.09
Atherosclerosis	42	48	0.72	0.46–1.13
Chronic coronary heart disease and angina pectoris	68	77	0.70	0.48–1.02
Myocardial infarction and severe coronary pathology	3	7	0.36	0.09–1.42

^a^OR = odds ratio,

^b^95% CI = 95% confidence interval.

## DISCUSSION

A number of studies on the biological effects of ionizing radiation have shown that radiation may damage DNA directly by energy transfer from particles to matter, or indirectly by forming a pool of free radicals [1, 19–21]. As mentioned previously, the telomere region is rich in guanine, which makes telomeres highly susceptible to oxidative damage [23], and exposure to ionizing radiation may affect telomere structure. However, currently available data on telomere length changes induced by ionizing radiation are ambiguous, with little evidence of an irradiation effect on telomere length regulation pathways [24–26].

A number of extrinsic factors are known to cause telomere shortening, but telomere lengthening after harmful exogenic exposure has been less investigated. The majority of studies on telomere length changes after irradiation have been conducted on animal models or in cell cultures shortly after exposure to high doses of ionizing radiation [37, 38], and late effects have generally not been evaluated. Human studies have been limited by small cohort size [27]. Due to large interindividual variability in telomere length in people of similar age and in different types of cells, there are certain difficulties with precise evaluation of telomere length measurements. Therefore, for an accurate assessment of the impact of ionizing radiation on telomere length, it may be necessary to increase the cohort size of the exposed subjects. The real-time q-PCR technique used in the present study has made possible effective and accurate measurement of a large number of samples and has revealed RTL differences between the subgroups under investigation, regardless of large interindividual variability.

In the current investigation, it has been possible to observe the situation in humans who have been exposed to small doses of ionizing radiation for a long time. The Latvian CNPP clean-up workers have been living in a relatively radioactively uncontaminated area since returning to Latvia. This fact allowed evaluation of the effect of prior ionizing radiation exposure on human health, using unexposed inhabitants of Latvia as controls. Moreover, the CNPP clean-up workers have been examined thoroughly a number of times, which provided the opportunity to evaluate telomere length changes and possible associations with a range of health issues. As mentioned earlier, data on documented irradiation doses in the CNPP clean-up workers is limited and unreliable for assessment of radiation effects because they were assessed on site in an inaccurate way that didn't take into account internal exposure during their stay in Chernobyl [7, 34, 35]. Hence, for more precise assessment of the impact of irradiation on telomeres, CNPP accident recovery operators were divided into several subgroups according to the characteristics of their work in Chernobyl. Our investigation of RTLs did not reveal any significant correlation of telomere length with recorded dose. This indicates that, in the case of the CNPP clean-up workers, the most reliable way to assess their exposure is through evaluation of tasks performed during their stay in Chernobyl together with year of participation.

It should be taken into account that more than 20 years have passed since direct contact with the radiation source was discontinued; therefore, a number of other factors might have influenced the results of telomere length measurements. The most important contributing factors might be smoking, alcohol consumption, chronic psycho–emotional stress, influence of toxic substances accumulated in the body during the stay in Chernobyl (e.g*.* heavy metals like lead), large number of chronic diseases in the one person at the same time, consequent treatment with a range of pharmaceuticals for a long period, as well as regular medical examinations (including X-ray). On the other hand, most of these conditions are known to be associated with telomere shortening.

Our investigation has revealed that CNPP clean-up workers do not have shortened telomeres; moreover, they have slightly longer telomeres than non-exposed people. Longer telomeres appear to be more common in CNPP clean-up workers who participated in 1986, as well as in those who performed so-called ‘dirty tasks’, such as deactivation of radioactively contaminated objects and digging of soil in Chernobyl and in the 30-km zone around Chernobyl. In other words, a tendency for elongation of telomeres was found in people who were more heavily exposed to ionising radiation. During their stay in Chernobyl, a certain level of radioactive substances accumulated in the body of recovery workers through inhaling the dust, ingesting local food and drinking contaminated water [7]. Workers who performed decontamination and digging tasks had a greater probability of incorporating larger amounts of radioactive isotopes into their bodies as a result of closer contact with contaminated objects.

The half-life of some radionuclides released into the environment after the Chernobyl disaster (such as ^137^Cs and ^90^Sr) is ∼30 years [7], and of others can be > 20 000 years [39]. Some of these radionuclides accumulate in bones (e.g. the retention half-time in the skeleton for strontium and uranium is >20 years) [39]. The decay of even a small amount of such long-living isotopes in human tissues might expose cells at close proximity (e.g. bone marrow stem cells) to a mixture of ionizing radiation of a range of types. The effect of such exposure may accumulate with time and cause alteration of metabolic processes and regulating pathways.

In workers who performed decontamination and digging tasks, telomere length had a slight tendency to increase with age; contrariwise, in the remainder of the workers, telomeres had a tendency to shorten with age, similarly to the control group. Deactivation and workers who performed digging tasks did dirtier and dustier work in Chernobyl and would have incorporated more radionuclides than other workers. More intensive long-term irradiation of cells in their body (e.g. bone marrow cells from radionuclides deposited in bones) could have activated telomerase gene expression mechanisms, which act to elongate telomeres. In those who performed other tasks, the level of activation of telomerase gene regulation mechanisms was probably less, and this might explain the similarity with the control group.

Significant shortening of telomeres occurs at two stages—early in childhood and after 60 years [40]. In our study, the age of participants was mainly between 40 and 70, so it is likely that more noticeable changes in telomere length will appear later, when these people get older. It was found that in the presence of certain disorders the RTL differed significantly between people with and without the particular diseases. It is important to note that the most elongated telomeres appeared in people with malignancies, but exclusion of them from analysis still revealed longer telomeres in the remaining CNPP clean-up workers compared with the control group, that is likely to be related to precancerous conditions in exposed people. On the other hand, it was evident from our results that aging-associated diseases, such as atherosclerosis, chronic coronary heart disease, senile cataract, osteoporosis and insulin-independent diabetes, were associated with shorter telomeres. This finding is consistent with other studies of telomere length evaluation in the presence of these disorders in non-irradiated humans [41–43]. The current work did not seek to evaluate the effect of severity, morphologic characteristics, or the stage of a particular disease on telomere length, although certain effects could well be discovered.

It is known that, as well as ionizing radiation, significant psycho–emotional stress and other factors (such as lead poisoning) occurred during the Chernobyl accident aftermath [7, 33]. The majority of the liquidators lived under chronic psycho–emotional stress for a long time after their return from Chernobyl [44]. Considering the large number of chronic disorders, chronic psycho–emotional stress, exposure to toxic substances, and long-term low-dose exposure to various types of ionizing radiation from incorporated long-living radionuclides, telomere length was expected to be shortened in CNPP clean-up workers. However, the results of our study did not demonstrate telomere shortening in CNPP recovery workers. The probable explanation for such a finding might be the high prevalence of malignancies and precancerous conditions among CNPP clean-up workers.

Ionizing radiation is known to be a proven carcinogen of the first group [45]. Our finding of long telomeres in CNPP clean-up workers after protracted exposure to ionizing radiation is consistent with the discovery by Li *et al.* (2012) of telomere elongation after chronic exposure (through drinking water) to such evidently carcinogenic substance as inorganic arsenic [46]. Recent studies made in cell cultures also discuss possible mechanisms of telomere lengthening following radiation exposure [47]. Several studies with cell lines have demonstrated that short telomeres increase sensitivity to radiation [48, 49]. It is likely that in the case of chronic exposure to radiation, human tissues develop defensive reactions against ongoing DNA damage. We suppose that one such reaction in the case of the CNNP clean-up workers might be upregulation of telomerase *hTERT* gene expression, leading to the elongation of telomeres. The combination of several conditions being present simultaneously (such as a specific mixture of regulating cytokines and a certain conformation of DNA telomeric ends) is needed for initiating upregulation of the telomerase gene [13, 14, 38, 50]. Cells chronically exposed to ionizing radiation might have a higher probability of fulfilling the conditions for initiation of telomerase activation. The telomere length of PBLs might indicate the general state of the regulating processes in an exposed organism. Such changes in non-cancer cells undergoing protracted irradiation might indicate a step towards malignant transformation. It appears likely that our finding of telomere elongation after protracted exposure to ionizing radiation indicates activation of telomerase as a chromosome healing mechanism following damage. On the other hand, it might reflect defects in telomerase regulation that could potentiate carcinogenesis. This is consistent with the suggestion of Lan *et al.* that individuals with longer telomeres in PBLs may have an increased risk of cancer development [51].

Considering the presence of multiple aging-associated diseases and frequently occurring malignancies in young CNPP clean-up workers [28 –31, 33], along with the absence of shortened telomeres in this group, the mechanism of regulation of senescence mechanisms in chronically irradiated people remains unclear. In studies of effects of radiotherapy on cancerous and healthy human cells, it was noticed that after irradiation with sublethal doses, both types of cells enter stress-induced premature senescence (SIPS). SIPS is not triggered by dysfunctional telomeres (as is usually seen in the case of replicative senescence), and it is not associated with telomere shortening [52, 53]. SIPS manifests as the arrest of cell growth and division with gradual cell senescence after non-lethal exposure of the cell to a harmful agent—such a reaction might develop as an alternative to cell apoptosis. Cells that undergo SIPS can remain alive for a longer time after exposure [52, 53]. In the case of CNPP clean-up workers, the SIPS mechanisms might be another adaptive response to protracted damaging irradiation.

In light of the higher incidence of cancers in irradiated people compared with the non-irradiated population [45], the telomere elongation observed in CNPP clean-up workers provides information on probable defects in the regulation of telomere length maintenance, particularly in the expression and activity of telomerase. Future work should therefore include evaluation of telomerase activity and telomerase expression regulating factors, as well as of the regulation of apoptotic processes in irradiated people.

## SUPPLEMENTARY DATA

Supplementary data is available at the *Journal of Radiation Research* online.

## FUNDING

This study was supported by The National Research Programme ‘Development of new prevention, treatment, diagnostics means and practices and biomedicine technologies for improvement of public health’ and by The European Social Fund project for doctoral study realization (agreement No. 2009/0147/1DP/1.1.2.1.2/09/IPIA/VIAA/009). Funding to pay the Open Access publication charges for this article was provided by the Institute of Occupational Safety and Environmental Health (Riga Stradins University).

## Supplementary Material

Supplementary Data
