# Supplementary material for: Telomere length in Chernobyl accident recovery workers in the late period after the disaster
Source: J Radiat Res. 2014 Jul 11;55(6):1089–100. doi: 10.1093/jrr/rru060 (PMC4229925; doi:10.1093/jrr/rru060)
Supplement: Supplementary Data [file supp_55_6_1089__index.html]

Telomere length in Chernobyl accident recovery workers in the late period after the disaster — Telomere length in Chernobyl accident recovery workers in the late period after the disaster — Supplementary Data 

# Telomere length in Chernobyl accident recovery workers in the late period after the disaster

## Supplementary Data

Supplementary Data

**Files in this Data Supplement:**

- Supplementary Data - Docx file
