# Supplementary material for: Telomere length in Chernobyl accident recovery workers in the late period after the disaster
Source: J Radiat Res. 2014 Jul 11;55(6):1089–100. doi: 10.1093/jrr/rru060 (PMC4229925; doi:10.1093/jrr/rru060)
Supplement: Supplementary Data [file supp_rru060_rru060supp.docx]

Supplemental material. Median age and prevalence of disorders in study population dividing by subgroups and comparing with general population of Chernobyl accident recovery workers from Latvia.

| **Characteristics** | **Study population of CNPP clean-up workers** | | | | | | | **General population of CNPP clean-up workers from Latvia*** |
| --- | --- | --- | --- | --- | --- | --- | --- | --- |
|  | **1986-year participants** | **1987-1991 year participants** | **Deactivation tasks** | **Other tasks** | **High risk** | **Low risk** | **Total** |  |
| Number of individuals in corresponding group | 306 | 271 | 231 | 250 | 332 | 252 | 595 | 3993 |
| Median age in years (IQR) at participation time in CNPP recovery works | 28 (24; 34) | 33 (29; 37) | 31 (26; 35) | 31 (25; 36) | 29 (24; 34) | 33 (29; 37) | 31 (25; 35) | 31 (26; 36) |
| Median age in years (IQR) at blood sampling time | 53 (48; 59) | 56 (52; 60) | 55 (50; 59) | 54 (49; 60) | 53 (49; 58) | 56 (53; 60) | 55 (50; 59) | 54 (49; 59) |
| Prevalence of disorders per 1000 persons (number of cases; median age at blood sampling time, (IQR)): | | | | | | | | |
| Malignant neoplasm | 92 (n=28; 59 (53; 63)) | 89 (n=24; 58 (56; 60)) | 87 (n=20; 58 (53; 61)) | 92 (n=23; 59 (56; 61)) | 93 (n=31; 58 (53; 62)) | 83 (n=21; 58 (56; 61)) | 87 (n=52; 58 (55; 62)) | 49 (n=196; 59 (54; 63)) |
| Insulin independent diabetes | 88 (n=27; 58 (53; 65)) | 107 (n=29; 61 (56; 64)) | 65 (n=15; 57 (52; 64)) | 132 (n=33; 61 (56; 65)) | 90 (n=30; 58 (52; 64)) | 103 (n=26; 62 (57; 64)) | 96 (n=57; 59 (54; 65)) | 61 (n=243; 57 (52; 61)) |
| Benign thyroid gland diseases | 503 (n=154; 54 (49; 59)) | 424 (n=115; 56 (52; 60)) | 533 (n=123; 55 (50; 59)) | 456 (n=114; 54 (51; 60)) | 494 (n=164; 54 (49; 59)) | 444 (n=112; 56 (52; 61)) | 466 (n=277; 55 (51; 60)) | 411 (n=1640; 55 (50; 59)) |
| Benign prostate hyperplasia | 154 (n=47; 59 (55; 62)) | 188 (n=51; 59 (55; 64)) | 195 (n=45; 59 (56; 61)) | 176 (n=44; 60 (54; 64)) | 154 (n=51; 59 (55; 61)) | 194 (n=49; 59 (56; 65)) | 168 (n=100; 59 (55; 63)) | 122 (n=486; 58 (53; 63)) |
| Osteoporosis | 62 (n=19; 58 (51; 65)) | 78 (n=21; 57 (54; 61)) | 61 (n=14; 58 (53; 62)) | 72 (n=18; 58 (53; 61)) | 54 (n=18; 59 (52; 64)) | 91 (n=23; 57 (53; 61)) | 69 (n=41; 58 (53; 62)) | 78 (n=310; 57 (52; 61)) |
| Senile cataract | 333 (n=102; 58 (53; 62)) | 435 (n=118; 59 (55; 61)) | 381 (n=88; 58 (54; 61)) | 392 (n=98; 58 (54; 63)) | 328 (n=109; 56 (53; 60)) | 444 (n=112; 59 (55; 62)) | 373 (n=222; 58 (54; 61)) | 268 (n=1072; 59 (54; 63)) |
| Atherosclerosis | 147 (n=45; 59 (55; 68)) | 162 (n=44; 60 (56; 62)) | 152 (n=35; 59 (56; 62)) | 168 (n=42; 61 (56; 66)) | 151 (n=50; 59 (55; 65)) | 159 (n=40; 60 (56; 66)) | 151 (n=90; 60 (56; 65)) | 116 (n=463; 58 (54; 63)) |
| Chronic coronary heart disease and angina pectoris | 229 (n=70; 59 (50; 63)) | 273 (n=74; 58 (54; 62)) | 286 (n=66; 58 (54; 62)) | 252 (n=63; 57 (52; 64)) | 235 (n=78; 57 (50; 63)) | 266 (n=67; 59 (54; 64)) | 245 (n=146; 58 (53; 63)) | 186 (n=741; 59 (54; 63)) |
| Myocardial infarction and severe coronary pathology | 13 (n=4; 61 (49; 62)) | 19 (n=5; 70 (61; 76)) | 17 (n=4; 63 (61; 72)) | 24 (n=6; 59 (54; 72)) | 12 (n=4; 61 (49; 62)) | 24 (n=6; 67 (57; 75)) | 17 (n=10; 62 (57; 71)) | 15 (n=60; 61 (56; 68)) |

* Data for alive on January 1^st^, 2011 CNPP clean-up workers from Latvia are shown (2171 out of 3993 participated in 1986, 1822 – in 1987-1991; 1694 performed deactivation tasks, 2299 – other tasks).

IQR – interquartile range (25^th^ and 75^th^ percentiles)
